# Predicting Ebola infection: A malaria-sensitive triage score for Ebola virus disease

**DOI:** 10.1371/journal.pntd.0005356

**Published:** 2017-02-23

**Authors:** Mary-Anne Hartley, Alyssa Young, Anh-Minh Tran, Harry Henry Okoni-Williams, Mohamed Suma, Brooke Mancuso, Ahmed Al-Dikhari, Mohamed Faouzi

**Affiliations:** 1 GOAL Global, Dublin, Ireland; 2 Faculty of Biology and Medicine, University of Lausanne, Lausanne, Switzerland; 3 Centre for Clinical Epidemiology, Institute of Social and Preventive Medicine, Lausanne, Switzerland; RTI International, UNITED STATES

## Abstract

**Background:**

The non-specific symptoms of Ebola Virus Disease (EVD) pose a major problem to triage and isolation efforts at Ebola Treatment Centres (ETCs). Under the current triage protocol, half the patients allocated to high-risk “probable” wards were EVD(-): a misclassification speculated to predispose nosocomial EVD infection. A better understanding of the statistical relevance of individual triage symptoms is essential in resource-poor settings where rapid, laboratory-confirmed diagnostics are often unavailable.

**Methods/Principal findings:**

This retrospective cohort study analyses the clinical characteristics of 566 patients admitted to the GOAL-Mathaska ETC in Sierra Leone. The diagnostic potential of each characteristic was assessed by multivariate analysis and incorporated into a statistically weighted predictive score, designed to detect EVD as well as discriminate malaria. Of the 566 patients, 28% were EVD(+) and 35% were malaria(+). Malaria was 2-fold more common in EVD(-) patients (p<0.05), and thus an important differential diagnosis. Univariate analyses comparing EVD(+) vs. EVD(-) and EVD(+)/malaria(-) vs. EVD(-)/malaria(+) cohorts revealed 7 characteristics with the highest odds for EVD infection, namely: reported sick-contact, conjunctivitis, diarrhoea, referral-time of 4–9 days, pyrexia, dysphagia and haemorrhage. Oppositely, myalgia was more predictive of EVD(-) or EVD(-)/malaria(+). Including these 8 characteristics in a triage score, we obtained an 89% ability to discriminate EVD(+) from either EVD(-) or EVD(-)/malaria(+).

**Conclusions/Significance:**

This study proposes a highly predictive and easy-to-use triage tool, which stratifies the risk of EVD infection with 89% discriminative power for both EVD(-) and EVD(-)/malaria(+) differential diagnoses. Improved triage could preserve resources by identifying those in need of more specific differential diagnostics as well as bolster infection prevention/control measures by better compartmentalizing the risk of nosocomial infection.

## Author summary

Four decades after the discovery of Ebola virus disease (EVD), the sources, reservoirs and dynamics of infection are still largely unknown and thus the threat of re-emergence remains ever present. As EVD thrives on fragile healthcare systems in the developing world, it is essential that triage tools are low-cost and easy-to-use in order to best allocate limited resources and ensure sustainability of EVD surveillance. From a public health perspective, sensitivity is paramount when screening for highly contagious and fatal diseases such as Ebola. However, once these suspect patients arrive at the treatment centres, specificity becomes far more important in order to accurately allocate them to risk-appropriate wards and better distribute limited resources. Currently, pre-test triage to identify “suspect” Ebola patients consists of a binary evaluation of non-specific symptoms that are shared by the much more prevalent disease: Malaria. Using these guidelines, over 70% of patients selected for admission to the potentially contagious environment of an ETC did not have Ebola. Within the ETC, patients may be further triaged into a higher risk “probable” ward on the basis of a clinically subjective assessment known as the “Ebola look”: since proven to have comparable accuracy to flipping a coin. While compartmentalising risk by stratification is an essential component to infection prevention and control measures, patient triage should be sufficiently accurate to justify to its benefit. This study constructs an easy-to-use and highly accurate (90%) triage scoring system that discriminates EVD infection risk in a malaria-sensitive manner: a strategy, which not only significantly improves the predictive accuracy for EVD but may also identify the (more deadly) infection of malaria.

## Introduction

Prior to the 2013–2015 epidemic of Ebola virus disease (EVD), fifteen outbreaks caused by the virulent *Zaire ebolavirus* strain had been recorded since the identification of the virus in 1976 [1]. The West African EVD epidemic started in December 2013, rapidly spreading from Guinea to Liberia and Sierra Leone to infect an estimated 28,600 people; over half of whom were in Sierra Leone [2]. Its unprecedented spread revealed a deadly potential to exploit weaknesses in public healthcare infrastructure [3], and established it as a disease for which low-income countries are at disproportionate risk [4]. As repeat outbreaks are predicted in this region for the near future [5], accurate, low-cost mechanisms to identify and triage EVD suspect cases are critical to ensure patient safety the sustainability of EVD surveillance.

Cumulatively, EVD outbreaks prior to 2013 affected less than 2400 people [1] and yielded limited systematic research on its diagnostic features. One of the more comprehensive studies during this time concluded that many of the differential diagnoses were clinically indistinguishable from Ebola without specific molecular testing [6]. This problem was inherited into the current WHO triage guidelines, which consist of a binary evaluation of non-specific symptoms that are shared by the much more prevalent disease, malaria [7]. Indeed, during the recent outbreak over 50% of “suspect” Ebola patients admitted to (the potentially contagious environment of) many ETCs did not have Ebola. From a public health perspective, sensitivity is paramount when screening for highly contagious and fatal diseases such as EVD, and specificity is often sacrificed in favour of a more sensitive detection. However, once these suspect patients arrive at the treatment centres, specificity becomes far more important in order to accurately allocate patients to risk-appropriate wards and better distribute limited resources. During the recent outbreak, patients admitted to the ETC were further triaged into a higher risk “probable” ward on the basis of a clinically subjective assessment known as the “Ebola look”: since proven to have comparable accuracy to flipping a coin [8, 9]. While compartmentalising risk by stratification is an essential component to infection prevention and control measures, patient triage should be sufficiently accurate to justify to its benefit.

Thus far, studies conducted on patient data from Ebola Treatment Centres (ETCs) in Sierra Leone, Guinea and Liberia have identified several clinical characteristics as being variably predictive of EVD diagnosis [9–18]. Patients that present with symptoms of confusion, conjunctivitis, intense fatigue, hiccups, vomiting [9], diarrhoea [9–11], and anorexia [14] have been noted as having a higher probability of EVD infection over other differential diagnoses. Some of these studies have shown that a combination of symptoms [9] or their inclusion in a disease score prediction model [11], is able to increase the odds of predicting EVD diagnosis. However, the variability across studies and their low positive predictive values show that further research is required before these strategies could be established as safe or effective triage techniques.

Malaria infection is not only a prevalent confounding diagnosis for EVD triage, but it was also shown to kill more people than EVD during the 2013 outbreak [19], which was likely due to its reduced prevention, diagnosis and treatment [20, 21]. Consequently, mathematical modelling has shown that the incidence of malaria infection during EVD is estimated to increase [21]. However, despite these statistics, no studies have adjusted the predictive values of individual symptoms according to their statistical association with malaria infection: a strategy, which may not only significantly improve their predictive accuracy for EVD but also possibly identify malaria infection.

In this retrospective cohort study, we analyse the clinical and epidemiological data of 566 patients admitted to the GOAL-Mathaska ETC in Port Loko, Sierra Leone. The diagnostic potential of each characteristic was analysed and incorporated into a statistically weighted and easy-to-use predictive score, designed to differentiate between EVD and malaria as well as greatly increase the specificity of EVD risk stratification whilst maintaining maximal detection sensitivity.

## Methods

### Ethics statement

Ethical approval for this research was granted by the Sierra Leone Ethics and Scientific Review Committee (SLESRC).

### Study design

This retrospective cohort study uses anonymized patient data collected between December 14, 2014 and November 15, 2015 at the GOAL-Mathaska ETC in Port Loko, Sierra Leone. Data comprised patient demographics, geographic location, clinical signs and symptoms, and laboratory results (a rapid diagnostic test for plasmodium infection and a semi-quantitative RTPCR for Ebola viremia, both performed at triage). We evaluate the potential of clinical characteristics to predict EVD diagnosis and use these results to construct a symptom-based diagnostic risk-stratification score, which corresponds to the predictive power of the most prevalent symptoms adjusted for the major differential diagnosis of malaria infection.

### Patient referral

The ETC was run by the humanitarian organization GOAL Global in cooperation with the Sierra Leonean Ministry of Health and Sanitation (MoHS). It opened in December 2014 and accepted 600 patients from a catchment area spanning 200km **(S1 Fig)**. On arrival at the ETC patients were allocated to “suspect” or “probable” wards according to the WHO guidelines [7]. Here, a “suspect” patient was selected for admission to the ETC based on the WHO guidelines which used various permutations of the following 3 elements: 1) acute fever, 2) contact history with an Ebola patient, and 3) any three of the following symptoms: headache, anorexia, lethargy, aching muscles, breathing difficulties, vomiting, diarrhoea, stomach pain, difficulty swallowing or hiccups (as summarised in **S2 Fig**). The distinction between “suspect” and the higher-risk category of “probable” was based on subjective clinical assessment and circumstantial epidemiological evidence, as per the WHO recommendation.

### Patient diagnosis, treatment and data collection

Blood was drawn from all patients on admission to the ETC and sent for Ebola virus testing at on-site laboratories managed by Public Heath England. An RDT malaria test was also performed at admission. Patients later testing positive for EVD by RT-PCR were transferred to the “confirmed” ward. All EVD(+) patients were treated according to standard treatment protocols developed by WHO and Médecins Sans Frontières [22, 23]. This included empiric antimalarial treatment, broad-spectrum antibiotics, and nutritional supplementation for all patients, as well as oral or intravenous fluid rehydration. Patients were discharged from the ETC only after returning two negative Ebola-specific RT-PCRs spaced 72 hours apart and the final decision was conditional to physician approval. Patients still meeting case definition after 2 negative test results were admitted for longer periods in order to account for possible delayed or prolonged symptom presentations **(S2 Fig)**.

### Data collection

The signs, symptoms and epidemiological data of each patient were recorded at triage by trained staff in a comprehensive and standardised questionnaire. Diagnosis was confirmed by semi-quantitative reverse transcriptase-PCR (RT-PCR) performed on the Cepheid GeneXpert instrument where the cycle threshold (Ct) value was used as an inverse proxy for viral load. Histidine-rich protein-II (HRP-II) antigen rapid diagnostic kits were used for the testing of malaria infection.

### Signs and symptoms

While symptoms were reported by the patient, haemorrhaging, pyrexia, and disorientation were recorded by clinicians after examination. Haemorrhagic signs included visible blood loss such as hematochezia, hematemesis, haematuria, epistaxis, haemoptysis or persistent haemorrhage from an IV catheter site as well as subcutaneous haemorrhage such as purpura and petechiae. Pyrexia was defined as a body temperature over 38°C, measured using an infrared thermal sensor. Disorientation was measured by trained ETC clinicians as per the AVPU alertness scale (where pain and unconsciousness were considered “disorientated”). Additionally, any specific mention of “confusion” or “disorientation” in the medical notes was also considered as positive for this variable.

### Cohorts and inclusion criteria

Of the 600 patients assessed, 10 were declared dead on arrival and 24 were classified as late transfers (treated elsewhere and thus convalescent on arrival) or had incomplete data. Thus, a total of 34 patients were excluded from this analysis. Of the 566 patients involved in the study, 100% had diagnostic test results for EVD, where, 27.5% tested EVD(+) (n = 158). 543/566 patients had malaria test results. The cohort was evaluated for missing values in each variable. Referral time (the time in days from symptom onset to admission at the ETC) had 20 cases of missing data. Further analysis was undertaken to evaluate the aetiology of missingness, which included demographic variables (such as age and sex), clinical severity variables (such as EVD viral load) as well as the covariates used in the final scoring model. Here, we found that subjects with missing data did not differ systematically from those with observed referral time, which is in favour of the hypothesis that the data were “missing completely at random”. In addition, we performed a sensitivity analysis using the “Hotdeck” imputation technique, which showed that the model coefficients did not change when using complete data [24].

### Data entry

To maximize data fidelity, patient files were entered into a secure Microsoft Excel database and cross-checked by 3 independent and trained analysts. Entry of clinical data was overseen by members of the clinical ETC staff. Graphs were constructed using GraphPad Prism, version 6.0. Univariate and multivariate analysis was conducted using STATA software, version 14 (StataCorp). Score validation was performed using “RMS” R-Package (R Development Core Team. ISBN 3-900051-07-0, URL: http://www.R-project.org). Results were deemed statistically significant at a p-value of less than 0.05.

### Primary data analysis

Epidemiological data and clinical variables were summarized by their frequencies and percentages. Univariate logistic regression was performed to assess the association between each predictor and the outcome of EVD diagnosis (reported as Odds-Ratios (OR) and p-values). Potential interactions were tested where the functional form of continuous variables (age and referral time) was checked using a fractional polynomial model [25]. The linearity assumption was confirmed for age but not for referral time . To simplify the triage score, referral time was coded into two categories ([4–9] days and [0–3] + [10–23] days). As there was an insufficient number of patients in the EVD(+) group (EVD(+) = 158, EVD(-) = 408) compared to the number of 29 potential predictors, only those associated to the outcome at a level of p<20% were considered into a Stepwise Backward selection procedure to fit a multivariable logistic regression model. Among the significant symptoms, those with the highest prevalence were favored for inclusion in the score. Model diagnostics was then performed to check for influential observations that impact coefficient estimates and a Hosmer-Lemeshow goodness-of-fit test was performed to assess calibration. Discriminative performance of the final model was assessed by calculating the Area Under the Receiver Operating Characteristics (ROC) Curve (AUC) and its 95% confidence interval. This value is a representation of the performance of a binary classifier system where the true positive rate (sensitivity) is plotted against the false-positive rate (1 − specificity). On this graph, perfect classification is represented by 100% area under the curve (AUC).

### Calculation of the triage score and model validation

The β-coefficient = log(OR) for each covariate of the final model was converted into an integer-based point-scoring system. The score was then derived as the sum of the covariates’ weighted scores. Internal validation using the bootstrap method (repeated 1,000 times) as described in Harrell et al. [26] was used to provide a more accurate estimate of the performance of the original model (AUC_original_). The algorithm allows calculating the optimism of the predictive discrimination in the original model. The difference (AUC_original_−optimism) gives the bootstrap-corrected performance of the original model.

### Secondary data analysis

In this analysis the outcome was a categorical dependent variable with three categories: 1) EVD(+)only, 2) Malaria(+)only, and, 3) Double negative (EVD(-)/Malaria(-)). To identify factors associated with the outcome, we performed a multinomial logistic regression analysis using the double negative group as a reference. Relative-Risk Ratios (RRR) and p-values were calculated to assess the strength of discrimination between the three categories.

## Results

### 1. Epidemiological characteristics of EVD admissions

**Fig 1:**
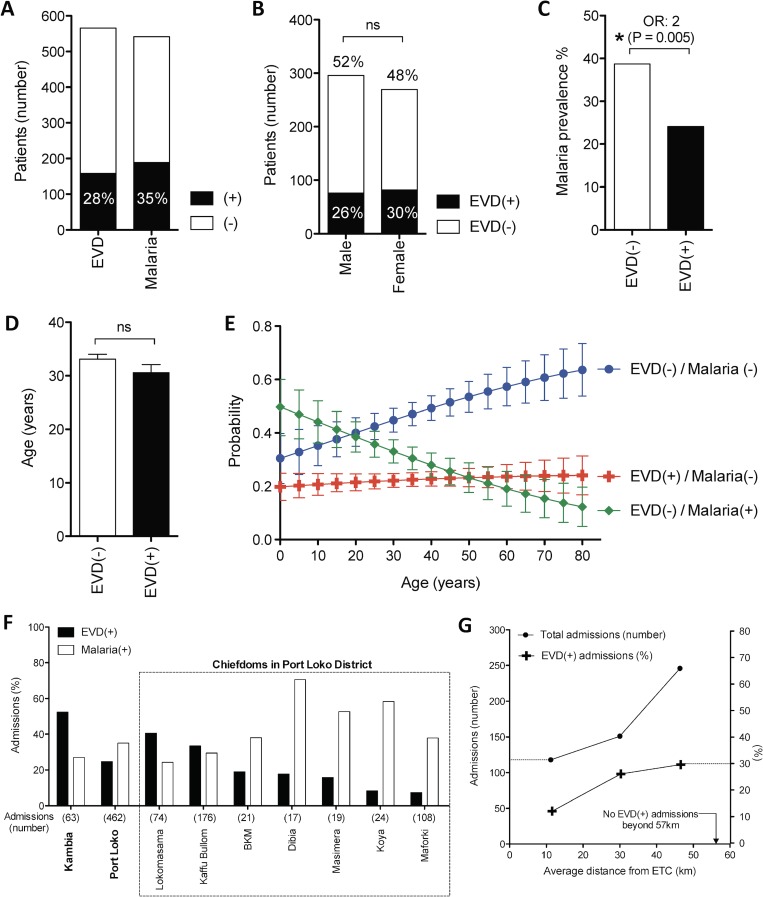
Demographic and epidemiological characteristics of EVD infection. **(A)** Number of patients according to EVD or malaria test result. **(B)** Gender distribution of EVD infection. **(C)** Malaria prevalence among EVD(-) and EVD(+) cohorts. **(D)** Average age of EVD(-) and EVD(+) cohorts. **(E)** Probability of testing EVD(+), EVD(-)/malaria(+) or EVD(-)/malaria(-) according to age. **(F)** Geographical distribution of EVD and malaria prevalence among admissions at the GOAL-Mathaska ETC, Sierra Leone^†^. **(G)** Number of admissions and EVD prevalence according to distance of the referred patient from the ETC^†^. ^†^ Representing 525/552 patients, for which EVD status and geographical location is known. *: p<0.05, **: p<0.005, ***: p<0.001, ns: not significant, ETC: Ebola Treatment Centre.

Of the 566 patients included in this study, 27.5% tested positive for EVD (n = 158). Malaria test results were available for 543 patients, of whom, 34.6% were positive (n = 188) **(Fig 1A)**. Gender was evenly distributed among admissions and there were no significant differences between EVD(+) and EVD(-) cohorts **(Fig 1B)**. Confirming its role as a major differential diagnosis, malaria infection was 2-fold more likely in the EVD(-) cohort than in the EVD(+) cohort (p = 0.005) **(Fig 1C)**. This quantifies the need for malaria-sensitive triage in order to better separate EVD(+) and EVD(-) patients. The mean age for all ETC admissions was 32.4 years, which was similar for EVD(+) and EVD(-) cohorts (30.6 vs. 33.1 years respectively) (**Fig 1D**). Indeed, probability of being infected with EVD did not vary with age **(Fig 1E)**, unlike malaria, which was more probable at younger ages **(Fig 1E)**. Oppositely, the probability of being neither EVD(+) nor malaria(+) increased with age, indicating a wider range of differential diagnoses among older patients **(Fig 1E)**.

Geographically, EVD and malaria prevalence was clustered in several locations across the catchment area of the GOAL-Mathaska ETC, where Kambia district had the highest percentage of EVD(+) cases among admissions **(Figs 1F and S1)**. These variations could be related to the physical distance of the referring centre from the ETC, where the percentage of EVD(+) admissions increased by over 20% with increasing distance **(Fig 1G)**.

### 2. Performance of current pre-EVD-test triage guidelines

**Fig 2:**
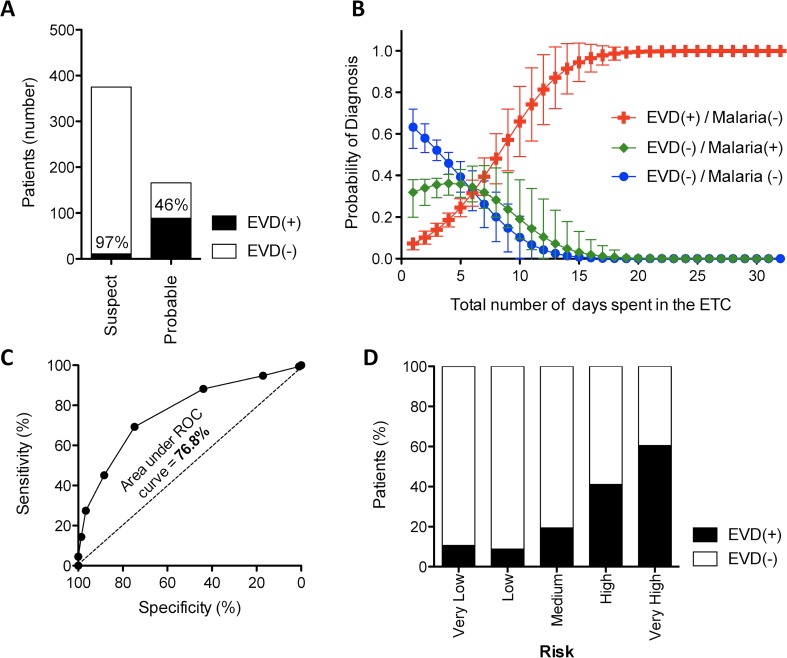
Accuracy of current triage methods. **(A)** Number of EVD(+) and EVD(-) patients triaged into the low-risk “suspect” and high-risk “probable” wards using the WHO triage protocol [7]. **(B)** Number of days spent in the ETC according to the probability of being diagnosed as either EVD(+) (red) or EVD(-) with malaria (green) or with neither EVD nor malaria (blue). **(C)** The sensitivity and specificity of predicting EVD(+) patients in our cohort using the scoring system of Levine et al. [11]. The area under the receiver-operator characteristic (ROC) curve represents the discriminative power of the score. **(D)** Percentage of EVD(+) and EVD(-) patients in our cohort classified in the various risk categories as proposed by the scoring system of Levine et al. [11].

According to the WHO guidelines [7], pre-EVD-testing triage of suspect Ebola cases took place in 2 stages **(S2 Fig)**. Firstly, patients were identified for admission to the ETC after meeting the symptomatic criteria of the case-definition. As shown in **Fig 1A**, 72.5% of all patients were incorrectly selected for admission into the ETC (i.e. later testing EVD(-)). The next stage of pre-EVD-test triage used clinical and epidemiological grounds to discriminate a higher risk “probable” group. While this process correctly identified 89% of all EVD(+) patients for allocation into the probable ward, 46% of selected patients in this high-risk ward later tested EVD(-) **(Fig 2A)**. Nevertheless, this process successfully reduced EVD(+) patients in the lower-risk “suspect” ward to 3% **(Fig 2A)**.

Once patients were admitted to the ETC, discharge was conditional on two EVD-negative test results spaced 72 hours apart in addition to clinical approval **(S2 Fig)**. Among the EVD(-) patients admitted to the ETC, the average number of days spent awaiting discharge approval was 12 hours longer for those infected with malaria (p = 0.045) **(Fig 2B)**.

A recent report by Levine et al. described an elegant diagnostic score to improve pre-test triage accuracy by combining the weighted points for EVD contact (+2), diarrhoea (+1.5), anorexia (+1), myalgia (+1), dysphagia (+1) and abdominal pain (-1) [11]. Using this algorithm, we were able to externally validate the relevance of the score on our cohort, obtaining an area under the ROC curve of 76.8% **(Fig 2C)** (almost identical to Levine et al., who obtained 75%). However, even with this risk stratification, the “very high” risk category still included over 40% EVD(-) patients **(Fig 2D)**, which would have been only a marginal improvement (<5%) on the current WHO criteria used for admission to the “Probable” ward **(S2 Fig)** [7].

### 3. Prevalence and diagnostic potential of clinical characteristics recorded at admission

**Fig 3:**
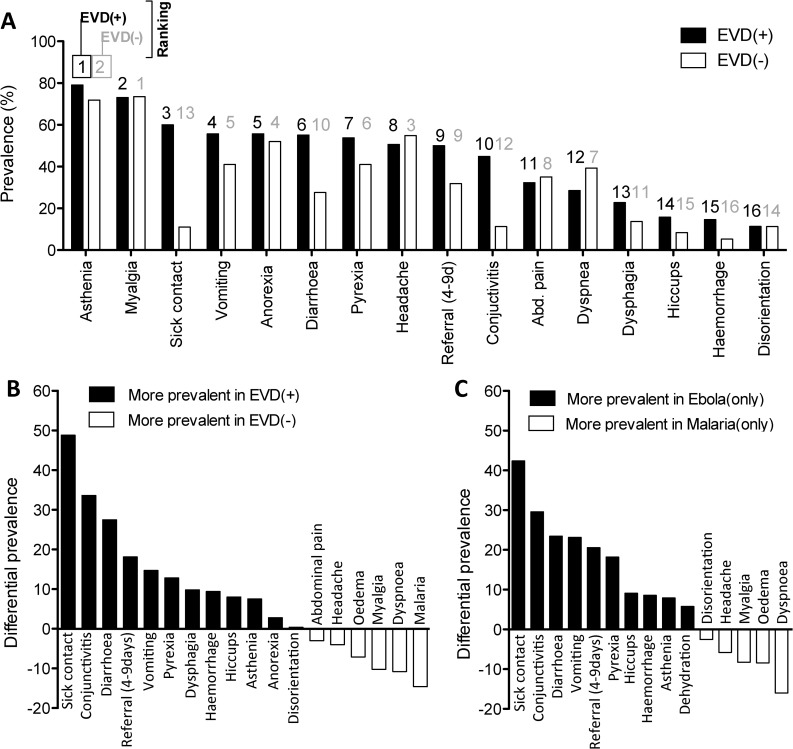
Prevalence of the clinical signs and symptoms recorded at triage. **(A)** Prevalence of triage symptoms for EVD(+) and EVD(-) cohorts ranked according to the prevalence in EVD(+). Rankings from 1–16 are listed above each bar: black for EVD(+) and grey for EVD(-). **(B)** Differences in symptom prevalence between EVD(+) and EVD(-) cohorts. Positive values are more prevalent in EVD(+) cases. Negative values are more prevalent in EVD(-) cases. **(C)** Differences in symptom prevalence between EVD(+)only patients and malaria(+)only patients. Positive values are more prevalent in EVD(+)only cases. Negative values are more prevalent in malaria(+)only cases. **EVD(+)only:** EVD(+)/malaria(-); **Malaria(+)only:** EVD(-)/malaria(+)

In an attempt to improve the accuracy of EVD(+) triage, we analysed the prevalence and diagnostic potential of the major clinical signs, symptoms and laboratory values among the EVD(+) and EVD(-) patients. Symptoms reported by over 50% of EVD(+) patients at triage were asthenia, myalgia, anorexia, vomiting, diarrhoea, pyrexia, and headache **(Fig 3A and Table 1)**. The prevalence of several triage symptoms was notably different between EVD(-) and EVD(+) patients, as can be seen by comparing their ranking **(Fig 3A)** or their differential prevalence **(Fig 3B)**. As expected, a history of possible “sick contact” with an EVD(+) patient was approximately 50% more common among those later diagnosed as EVD(+). Further, 20% more EVD(+) patients reported to the ETC within 4–9 days of their first symptom compared to their EVD(-) counterparts. The clinical features of conjunctivitis and diarrhoea, vomiting and pyrexia were over 10% more prevalent in EVD(+) patients at triage. Oppositely, malaria infection, dyspnoea and myalgia were over 10% more prevalent in EVD(-) patients **(Fig 3B)**.

**Table 1:** Univariate and multivariate logistic regression analysis for the diagnostic potential of triage characteristics: EVD(+) vs. EVD(-).

	Prevalence				Univariate		Multivariate			
	EVD(-)		EVD(+)		EVD(+) diagnosis		EVD(+) diagnosis		Score weighting	
	%	*(n)*	%	*(n)*	OR	*P value*	OR	*P value*	Coeff.	Weight^‡^
**Clinical characteristics† reported at triage**										
**TOTAL**	72.1	*(408)*	27.9	*(158)*	-		-			
**Sick contact**	11.3	*(44)*	60.1	*(92)*	12.0	*0*.000*	19.4	*0*.000*	3.0	**+ 6**
**Conjunctivitis**	11.5	*(47)*	44.9	*(71)*	6.3	*0*.000*	7.2	*0*.000*	2.0	**+ 4**
**Diarrhoea**	27.2	*(111)*	55.1	*(87)*	3.2	*0*.000*	3.8	*0*.000*	1.3	**+ 3**
**Ref time** (4–9days)	31.9	*(109)*	50.0	*(69)*	2.1	*0*.000*	3.7	*0*.000*	1.3	**+ 3**
**Vomiting**	41.7	*(170)*	55.7	*(88)*	1.8	*0*.003*	-			
**Pyrexia** (>38°C)	40.9	*(167)*	53.8	*(85)*	1.7	*0*.006*	1.8	*0*.044*	0.6	**+ 1**
**Dysphagia**	13.0	*(53)*	22.8	*(36)*	2.0	*0*.005*	2.2	*0*.034*	0.8	**+ 2**
**Haemorrhage**	5.2	*(21)*	14.6	*(23)*	3.1	*0*.000*	2.9	*0*.036*	1.1	**+ 2**
**Hiccups**	7.8	*(32)*	15.8	*(25)*	2.2	*0*.005*	-			
**Asthenia**	71.6	*(292)*	79.1	*(125)*	1.5	*0*.*069*	-			
**ORL haemorrhage**	0.3	*(1)*	7.6	*(12)*	33.5	*0*.001*	-			
**Dehydration**	9.6	*(39)*	13.9	*(22)*	1.5	*0*.*135*	-			
**Hematochezia**	2.0	*(8)*	5.1	*(8)*	2.7	*0*.*054*	-			
**Anorexia**	52.9	*(216)*	55.7	*(88)*	4.5	*0*.041*	-			
**Anuria**	1.0	*(4)*	3.2	*(5)*	3.3	*0*.*078*	-			
**Haematuria**	0.3	*(1)*	1.3	*(2)*	5.2	*0*.*179*	-			
**Disorientation**	11.0	*(45)*	11.4	*(18)*	1.0	*0*.*902*	-			
**Hepatomegaly**	2.9	*(12)*	3.2	*(5)*	1.1	*0*.*889*	-			
**Rash**	2.9	*(12)*	2.4	*(4)*	0.9	*0*.*792*	-			
**Haemoptysis**	1.5	*(6)*	0.6	*(1)*	0.4	*0*.*432*	-			
**Abdominal pain**	35.3	*(144)*	32.3	*(51)*	0.9	*0*.*498*	-			
**Headache**	54.7	*(223)*	50.6	*(80)*	0.9	*0*.*389*	-			
**Myalgia**	73.5	*(300)*	63.3	*(100)*	0.6	*0*.017*	0.5	*0*.012*	-0.8	**- 2**
**Dyspnoea**	39.0	*(159)*	28.5	*(45)*	0.6	*0*.020*	-			
**Laboratory results at triage**										
**Malaria infection**	38.7	*(154)*	24.1	*(35)*	0.5	*0*.001*	-			
**Demographic characteristics**										
**Age** (mean(SD))	33.2	*(18*.*4)*	30.6	*(19)*	1.0	*0*.*140*	-			
**Sex** (female)	46.0	*(188)*	51.9	*(82)*	1.3	*0*.*214*	-			

†Characteristics appear in order of their differential prevalence (EVD(+)—EVD(-)).

The “Univariate” column shows the unadjusted OR of each characteristic to EVD infection (shaded with a heat map identifying the most predictive characteristics).

The “Multivariate” column presents only the characteristics used in the triage score. Coefficients (Coeff) and their mathematically manipulated score weightings are shown in the final column.

‡ Score weights are calculated as 2 X coefficient, rounded off to the nearest whole integer.

*: p<0.05, SD: standard deviation, OR: Odds ratio.

Univariate logistic regression revealed several signs and symptoms that were strongly predictive for the diagnosis of EVD and statistical significance was generally found among characteristics with the highest differential prevalence, such as sick contact, conjunctivitis, diarrhoea, referral time of 4–9 days, pyrexia, dysphagia, haemorrhage and hiccups (p<0.05 for all) **(Table 1)**. Oppositely, we found the strongest predictors for not having EVD were myalgia, dyspnoea and malaria infection (p<0.05 for all) **(Table 1)**. Indeed, malaria infection is a prevalent differential diagnosis of EVD manifesting with many of the same symptoms and may play a major role in reducing the level of triage accuracy [8].

In an attempt to better discriminate between the symptoms defining EVD and malaria, we analysed the differential prevalence and predictive potential of symptoms between EVD(+)/malaria(-) and EVD(-)/malaria(+) patient cohorts. Here, we identify several of the most predictive triage symptoms for malaria, such as dyspnoea, oedema, myalgia, and disorientation, which are thus poor indicators for EVD in a malaria endemic region **(Fig 3C and Table 2)**. Univariate analysis on the predictive value of these symptoms identified conjunctivitis, diarrhoea, vomiting, pyrexia, hiccups and haemorrhage as the strongest differential indicators for EVD infection in a malaria-endemic region (p<0.05 for each) **(Table 2)**.

**Table 2:** Multinomial univariate logistic regression analysis for the diagnostic potential of triage characteristics: EVD(+)only vs. Malaria(+)only vs. Double-negative control.

	Prevalence						Univariate					
	(1)		(2)		(3)		(2 vs. 1)		(3 vs. 1)		(3 vs. 2)	
	Double-negative *(control)*		Malaria (+) only		EVD(+) only		Malaria(+) diagnosis		EVD(+) diagnosis		EVD(+) diagnosis	
	%	*(n)*	%	*(n)*	%	*(n)*	RRR	*P value*	RRR	*P value*	RRR	*P value*
**Clinical characteristics† reported at triage**												
**TOTAL**	47.4	*(237)*	30.6	*(153)*	22.0	*(110)*	-		-		-	
**Sick contact**	12.8	*(29)*	8.9	*(13)*	55.1	*(59)*	0.6	*0*.*233*	8.4	*0*.000*	52.4	*0*.000*
**Conjunctivitis**	12.2	*(29)*	9.2	*(14)*	41.8	*(46)*	0.7	*0*.*344*	5.2	*0*.000*	33.3	*0*.000*
**Diarrhoea**	27.4	*(65)*	25.3	*(39)*	50.9	*(56)*	0.9	*0*.*673*	2.7	*0*.000*	17.4	*0*.000*
**Vomiting**	35.0	*(83)*	53.6	*(82)*	58.2	*(64)*	2.1	*0*.000*	2.6	*0*.000*	0.5	*0*.*461*
**Ref. time** (4–9days)	33.7	*(66)*	29.9	*(40)*	54.3	*(51)*	0.8	0.465	2.3	*0*.001*	13.4	*0*.000*
**Pyrexia** (>38°C)	35.4	*(84)*	46.4	*(71)*	53.6	*(59)*	1.6	0.031*	2.1	*0*.001*	1.3	*0*.*248*
**Hiccups**	7.2	*(17)*	9.2	*(14)*	16.4	*(18)*	1.3	*0*.*482*	2.5	*0*.010*	3.0	*0*.*081*
**Haemorrhage**	5.1	*(12)*	4.6	*(7)*	13.6	*(15)*	0.9	*0*.*827*	3.0	*0*.008*	6.3	*0*.012*
**Asthenia**	73.0	*(173)*	69.9	*(107)*	80.9	*(89)*	0.9	*0*.*512*	1.6	*0*.*112*	4.0	*0*.045*
**ORL haemorrhage**	0.4	*(1)*	0.0	*(0)*	7.3	*(8)*	-	-	18.5	*0*.006*	-	*-*
**Dehydration**	9.7	*(23)*	10.6	*(16)*	15.5	*(17)*	1.1	*0*.*809*	1.7	*0*.*122*	1.4	*0*.*230*
**Dysphagia**	14.4	*(34)*	10.5	*(16)*	20.0	*(22)*	0.7	*0*.*264*	2.5	*0*.*185*	4.57	*0*.032*
**Anorexia**	49.0	*(116)*	57.5	*(88)*	52.7	*(58)*	1.4	*0*.*098*	1.2	*0*.*739*	0.6	*0*.*441*
**Anuria**	1.3	*(3)*	0.0	*(0)*	3.6	*(4)*	-	*-*	2.9	*0*.*162*	-	*-*
**Headache**	51.5	*(112)*	59.5	*(91)*	53.6	*(59)*	1.4	*0*.*122*	1.1	*0*.*708*	0.9	*0*.*345*
**Haematuria**	0.4	*(1)*	0.0	*(0)*	1.8	*(2)*	-	*-*	4.4	*0*.*230*	-	*-*
**Hematochezia**	2.5	*(6)*	0.7	*(1)*	2.7	*(3)*	0.3	*0*.*206*	1.1	*0*.*915*	1.6	*0*.*212*
**Haemoptysis**	0.8	*(2)*	2.0	*(3)*	0.9	*(1)*	2.4	*0*.*352*	1.1	*0*.*951*	0.5	*0*.*502*
**Abdominal pain**	34.6	*(82)*	36.0	*(55)*	34.6	*(38)*	1.1	*0*.*785*	1.0	*0*.*992*	0.1	*0*.*815*
**Hepatomegaly**	3.0	*(7)*	3.3	*(5)*	2.7	*(3)*	1.1	*0*.*861*	0.9	*0*.*907*	0.1	*0*.*801*
**Rash**	3.4	*(8)*	2.6	*(4)*	2.7	*(3)*	0.8	*0*.*672*	0.8	*0*.*749*	0.0	*0*.*955*
**Jaundice**	2.5	*(6)*	0.0	*(0)*	0.0	*(0)*	-	*-*	-	-	-	-
**Disorientation**	12.6	*(30)*	8.5	*(13)*	10.0	*(11)*	0.6	*0*.*203*	0.9	*0*.*476*	0.2	*0*.*676*
**Myalgia**	75.5	*(179)*	71.2	*(109)*	67.3	*(74)*	0.8	*0*.*347*	0.7	*0*.*109*	0.5	*0*.*490*
**Oedema**	8.4	*(20)*	5.2	*(8)*	0.0	*(0)*	0.6	*0*.*234*	-	-	-	-
**Dyspnoea**	46.0	*(237)*	29.4	*(45)*	30.0	*(110)*	0.5	*0*.001*	0.5	*0*.005*	0.0	*0*.*918*
**Demographic characteristics**												
**Age** (mean(SD))	36.0	*(17*.*1)*	28.0	*(18*.*6)*	32.1	*(18*.*8)*	1.0	*0*.000*	1.0	*0*.*066*	2.03	*0*.*154*
**Sex** (female)	12.8	*(29)*	8.9	*(13)*	55.1	*(59)*	1.2	0.282	1.5	*0*.*078*	0.55	*0*.*460*

**†**Characteristics appear in order of their differential prevalence (EVD(+)only—Malaria(+)only).

The “Univariate” columns show the unadjusted RR of each characteristic for the comparisons indicated (shaded with a heat map identifying the most predictive characteristics).

*: p<0.05, SD: standard deviation, RRR: relative risk ratio.

**EVD(+)only:** EVD(+)/malaria(-), **Malaria(+)only:** EVD(-)/malaria(+), **Double-negative:** EVD(-)/malaria(-).

### 4. Impact of EVD on time taken to report symptoms

**Fig 4:**
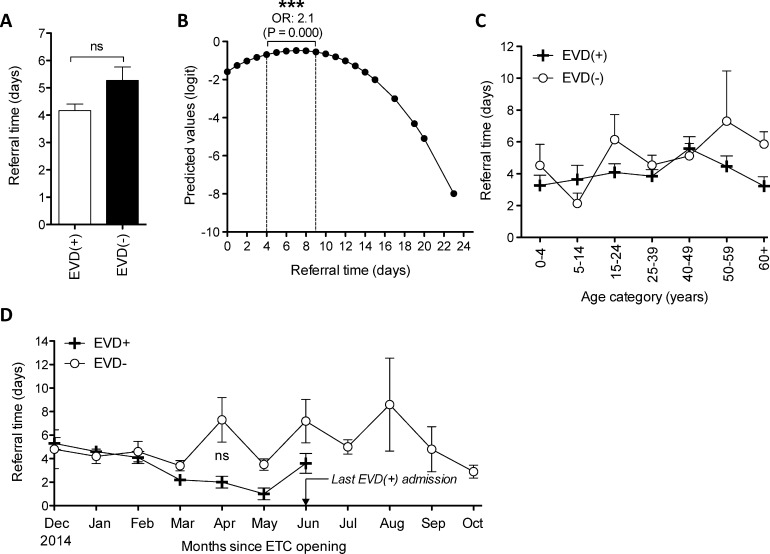
Impact of EVD on referral sensitivity. **(A)** Mean referral time (days since symptom onset at triage) for EVD(+) and EVD(-) cohorts. **(B)** Fitted relationship between referral time and outcome using fractional polynomial analysis [25]. **(C)** Mean referral time for EVD(+) and EVD(-) patients according to age categorisation. **(D)** Mean referral time for EVD(-) and EVD(+) patients over the entire time course of the study (December 2014 to October 2015). *: p<0.05, **: p<0.005, ***: p<0.001, ns: not significant, ETC: Ebola Treatment Centre.

The number of days from symptom onset to admission at the ETC (i.e. “referral time”) was available for 87.3% of the EVD(+) cohort and 83.9% of the EVD(-) cohort. The mean number of days from symptom onset to admission did not differ significantly between EVD(+) and EVD(-) cohorts (4.2 days vs. 5.3 days respectively, p = 0.16) **(Fig 4A)**. However, EVD(+) patients were 2.1 fold more likely to report to an ETC 4–9 days from symptom onset (p<0.0001) **(Fig 4B)**. Overall, gender and age were not significant factors in the time taken for a patient to present at an ETC. Referral time across age groups is shown in **Fig 4C**. We next investigated whether referral distance affected referral time. Comparing patients from the Port Loko and Kambia districts (average distances from the ETC are 27.1 and 40.0 km respectively), we found no significant difference in referral times. Temporal analysis showed that referral sensitivity improved among the EVD(+) cohort as the epidemic progressed **(Fig 4D)** until June 2015, when the last positive EVD case was admitted to the ETC (albeit not significantly different from EVD(-)).

### 5. Derivation of a malaria-sensitive triage scoring system for EVD

**Fig 5:**
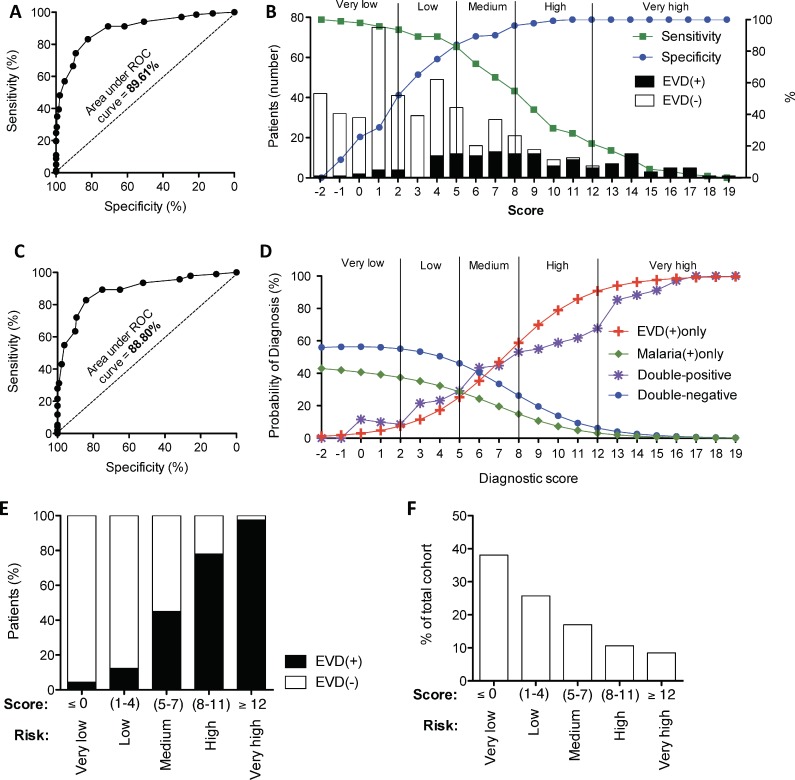
Derivation of a malaria-sensitive triage scoring system for EVD. The sensitivity and specificity of predicting EVD(+) patients in our cohort using the triage scoring system developed from the multivariate analysis of groups comparing **(A)** EVD(+) vs. EVD(-) and **(C)** EVD(+)/malaria(-) vs. EVD(-)/malaria(+). The area under the receiver-operator characteristic (ROC) curve represents the discriminative power of each score. **(B)** Sensitivity (green) and specificity (blue) according to the 22 score points. Prevalence of EVD(+) and EVD(-) patients are displayed as bar graphs and risk category cut-offs are shown as vertical lines. **(D)** Probability of being diagnosed as either EVD(+)only (red), Malaria(+)only (green) or double-negative (blue) according to the 22 points of the triage score. **(E)** Percentage of EVD(+) and EVD(-) patients classified in each risk category. **(F)** Percentage of the cohort captured in each risk category.

Performing multivariate analysis, we selected the clinical characteristics most predictive for EVD infection when comparing EVD(+) vs. EVD(-) as well as when comparing EVD(+)/malaria(-) vs. EVD(-)/malaria(+) **(Tables 1 and 2)**. By stepwise backwards elimination, and prioritizing the most prevalent symptoms, we identified 8 characteristics which yielded significant predictive values in both comparison groups. Characteristics that were statistically significant predictors of EVD infection were sick contact, conjunctivitis, diarrhoea, a referral time of 4–9 days, haemorrhage, dysphagia and pyrexia (p<0.05 for all). Additionally, we selected myalgia, as a significant negative predictor of EVD infection. We then calculated weightings from their predictive coefficients with the aim to find a simplified scoring model using whole integers and calculations limited to subtraction or addition. Testing the sensitivity and specificity of these weightings for the prediction of EVD infection, we found that the characteristics yielded an area under the ROC curve (AUC) of approximately 90% (89.61% for the comparison between EVD(+) vs. EVD(-) (CI95%: 86%, 93%) **(Fig 5A)** and 88.80% for the comparison between EVD(+)only vs. malaria(+)only (CI95%: 84%, 93%) **(Fig 5C)**). The risk category cut-offs are illustrated in **Fig 5B** and each category contains at least 10% of the cohort. **Fig 5D** shows that the selected variables and cut-offs not only discriminate between EVD(+)only and double-negative patients but also between EVD(+)only and malaria(+)only patients. Further, our score predicts double-positive patients similarly to EVD(+)only patients **(Fig 5D)**. Examining the accuracy of the score on our cohort, we found that the “very high” classification was able reduce the EVD(-) patients in the high-risk group to less than 3% **(Fig 5E)**. Further, the “high” risk category contained 80% correctly classified EVD(+) patients (>95% specificity) **(Fig 5E)**. At the other end of the scale, the “very low” risk category contained over 95% EVD(-)patients **(Fig 5E)** and represented approximately 40% of the total cohort **(Fig 5F)**. A table listing the full details and intercept of the multivariate analysis is available in the supplement **(S1 Table)**. An internal validation of the score to discriminate EVD(+) from EVD(-) samples yielded a final discriminative power of 88.73% **(Table 3)**.

**Table 3:** Internal validation of EVD triage score.

AUC_original_	Optimism	AUC_corrected_
89.61%	0.088%	88.73%

While a referral time of 4–9 days was significantly predictive of EVD diagnosis over the entire timeframe of the study **(Fig 4B)**, we tested the performance of our scoring system on patient populations arriving before and after this threshold and found minimal changes to sensitivity and specificity **(S3 Fig)** where our score maintained an AUC of over 85%.

**Fig 6:**
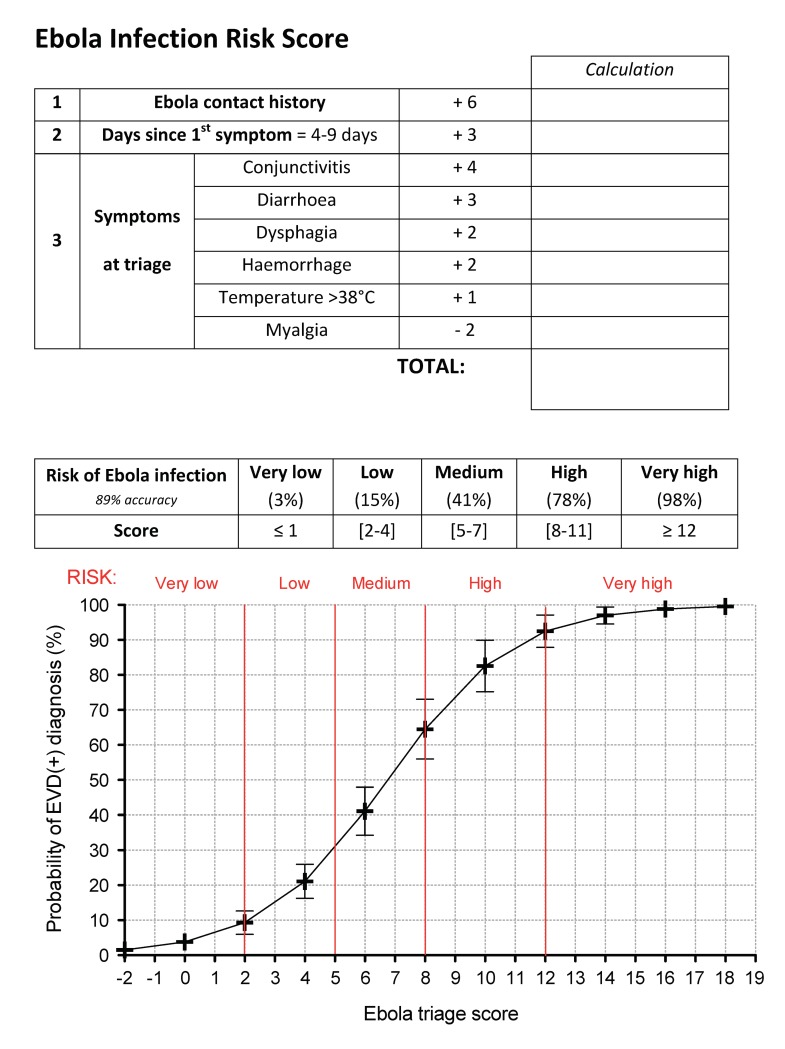
Scorecard to extrapolate the Ebola infection risk at triage.

As our scoring system is designed to be sensitive to endemic malaria, another potential limitation is that it may not work well on co-infected EVD(+)/malaria(+) patients. However, testing the score on co-infected patients within our cohort, we maintain an AUC of 91% (CI95%: 85.9%, 96.7%) for discrimination of EVD infection (i.e. no change) **(Table 4)**. An additional temporal concern would be malaria seasonality. However, testing scoring accuracy on the population presenting to the ETC during the low malaria transmission months (November to April) showed that the discriminative power remained within 3% of the overall value (85.56% AUC). As anticipated, this malaria-sensitive score was more powerful during the malaria season (98.55% AUC) **(S4 Fig)**. Tweaking the period considered as “high malaria transmission” by a month in either direction had no statistical effect. A printable template of the scoring system is found in **Fig 6**, including a probability curve on which to extrapolate the risk of EVD infection.

**Table 4:** Performance of EVD triage score on various populations.

Population	AUC (discriminative power)
Malaria(+)/Ebola(+)	91.02%
In malaria season	98.55%
Out of malaria season	85.90%
Out of peak referral time (4–9 days)	85.30%
Overall	89.61%

## Discussion

During the 2013 Ebola outbreak, the lack of specificity of pre-test triage overwhelmed Ebola treatment centres with inaccurately selected patients. Indeed, over 70% of patients selected for admission to the ETC of this study were EVD(-). As the aim of an ETC is to concentrate and isolate Ebola infection, it is a high-risk zone for which the benefits of admission must be carefully measured. Miscategorisation of EVD(-) individuals at triage can expose them to nosocomial EVD infection [27] as well as increasing sample-handling and thus the risk of contamination [28]. Unnecessary ETC admissions also cause significant physiological stress and social stigmatisation [29] as well as potentially reducing public compliance for symptom reporting [28]. Risk stratification amongst EVD suspect patients within the ETC, is a key element of infection prevention and control (IPC), which can compartmentalise risk by physically separating patients into risk-appropriate wards. However, the WHO triage protocol for this process gave little guidance to clinicians **(S2 Fig)**; and almost half of the individuals allocated to the resultant high-risk “probable” ward in this study were EVD(-). This is a result comparable with other ETCs [27, 30]. Further, it is important to remember that despite being EVD(-), all ETC admissions were selected on the basis of being unwell and thus better classifying those at lower risk of EVD infection may identify a target group that requires more in-depth differential diagnostics [8].

While the above studies raise concerns about the specificity of the WHO triage system, previous studies have identified issues about its sensitivity: a group who retrospectively applied the WHO case definition on patients from a previous epidemic, revealed that it displayed only 58% sensitivity and concluded that it was more suited to detect Marburg infection [31]. Performance of this triage protocol during the 2013 outbreak may have under-performed as it was not specifically developed for detection of the epidemic Makona strain of the Ebola virus: for which certain haemorrhagic signs were less common [32]. Indeed, despite Ebola’s notoriety as a “haemorrhagic disease”, haemorrhage was only seen in around 10% of our patients (albeit remaining a potently specific predictor of infection). Another issue which diluted the specificity of the WHO triage protocol, was the presence of malaria. Across West Africa and many other African Ebola outbreaks, malaria infection has been an important differential diagnosis. This was especially true for the younger age groups in our cohort, where up to age 45, patients were more likely to be infected with malaria alone than EVD (**Fig 1E, green line)**. As over 80% of the Sierra Leonean population is classified in this age group [33], it is clear that malaria represents a particularly prevalent differential diagnosis. Further, we show that EVD(-) patients who were infected with malaria were more likely to spend a longer period in the ETC **(Fig 2B)**. This is perhaps because discharge was conditional to the patient no longer meeting symptomatic EVD case-definition: a classification that has particular overlap with malaria. Taken together, this reveals that the inability to discriminate between malaria and EVD in triage poses a potentially important risk of nosocomial EVD infection.

The aim of this study was to identify clinical characteristics that could better discriminate between EVD(+) and EVD(-) as well as differentiate EVD from malaria. The prevalence of the various symptoms with which EVD(+) patients presented at triage was similar to other studies on cohorts in Sierra Leone, Liberia, and Guinea [9–18], where asthenia [13, 14], myalgia [14, 34], vomiting [9, 10, 12, 34], anorexia [10, 12, 34], diarrhoea [10, 12, 13, 34], pyrexia [9, 10, 13], and headache [10, 13, 14, 34] were the most common complaints **(Fig 3)**. However, evaluating the differential prevalence (for EVD(+) vs. EVD(-) and EVD(+)only vs. malaria(+)only) we found that asthenia was less than 10% more common in EVD(+) cases and that myalgia and headache were actually more common in EVD(-) and malaria(+)only than their EVD(+) counterparts. Characteristics with the highest differential prevalence were also the most statistically significant discriminators of EVD, namely, sick contact, conjunctivitis, diarrhoea, a referral time of 4–9 days, vomiting, pyrexia, dysphagia, haemorrhage and hiccups **(Tables 1 and 2)**. Further, multivariate analysis identified a combination of symptoms highly predictive for EVD infection. A statistically weighted score including conjunctivitis, diarrhoea, dysphagia, haemorrhage, fever, as well as the time taken to present the symptoms and Ebola contact history amounted to a 90% power to discriminate between EVD(+) and EVD(-) cases as well as between EVD(+) and EVD(-)/malaria(+), whilst still accurately identifying EVD(+)/malaria(+) co-infection (91% AUC) **(Fig 5)**.

As resources and available beds can become limited during a high-transmission period, it is crucial to provide a score that delineates multiple levels of risk and gives clinicians the power to decide the sensitivity limits of the triage to better adapt their available resources to the changing dynamics of an outbreak.

### Limitations

Various biases plague patient-reported data, where patients may deny EVD contact or misremember the date of symptom onset: all concerns raised previously by similar reports [9]. In our study, a referral time of 4–9 days was a highly significant discriminator between EVD(+) cases and both EVD(-) and malaria(+)only patients. Referral time may be particularly prone to socioeconomic nuance as it is inextricably linked to healthcare seeking behaviour. However, a systematic study on 4,437 cases of Ebola transmission in Liberia, showed no significant differences in referral time or hospitalisation access across socioeconomic strata [35]. Further, we show that there were no significant differences in referral time between genders or among different age groups **(Fig 4C)**. The referral time of 4–9 days was significantly predictive of EVD diagnosis over the entire timeframe of the study **(Fig 4B)** and testing the performance of our scoring system on patient populations arriving before and after this threshold resulted in minimal changes to sensitivity and specificity **(S3 Fig)**. Some differences in reporting behaviour do exist however. For example, it has been previously shown that adults in Sierra Leone have a significantly higher incidence of reporting possible EVD infection as compared to children [36]. In this study, we have a similar finding but show that the probability for receiving an EVD(+) test result was similar across all ages **(Fig 1E red line)**. This is explained by our observation that older patients were more likely to report to ETC with symptoms unrelated to EVD **(Fig 1E, blue line)**.

As our scoring system is based on its malaria-sensitive discrimination of EVD(+) patients, a potential limitation is that it may not work well on co-infected EVD(+)/malaria(+) patients. However, testing the score on co-infected patients within our cohort, we maintain an AUC of 91% (CI95%: 85.9%, 96.7%) for discrimination of EVD infection (i.e. no change) **(Table 4)**. An additional concern about a malaria-integrative score would be changing accuracy with malaria seasonality. However, testing scoring accuracy during the low malaria transmission months (November to April) also showed no significant difference in the discriminative power compared to the general population. As anticipated, this malaria-sensitive score performed better during the malarial transmission months of West Africa (May to October). Here, the power to discriminate between groups increased by 9% compared to the overall population (99% vs. 90%): a welcome deviation, considering the potential confusion that malaria may cause to triage **(S4 Fig)**. Importantly, our ETC opened in December 2014 and the last EVD(+) patient was admitted to our facility at the end of June 2015. Thus, the EVD(+) cohort is not fully represented across both seasons.

Despite this high performance, the true accuracy of any scoring system can only be tested and improved by external validation on large independent cohorts, which pool statistics to fine-tune the weightings and ensure the most generalizable application. Indeed, as with any cohort study, the generalizability is often limited to the geographic and demographic profile of the selection criteria. In an effort to test the generalizability of this cohort, we externally validated the triage scoring system proposed by Levine et al. [11]: a scoring system developed for a rural cohort in Liberia. Here, our results differed by less than 2%, and served to validate the representational capacity of our cohort as well as display the robustness of using such scoring systems across geographically disparate areas with socioeconomic nuance and variable malaria prevalence.

## Conclusion

This study identifies several clinical characteristics, which are significantly predictive for the diagnosis of EVD infection and proposes a highly specific malaria-sensitive triage tool capable of stratifying the risk of EVD, which may significantly increase the accuracy of pre-test EVD triage.

### Perspectives

As previously stressed, external validation and systematic meta-analyses are needed to fine-tune the statistical weightings of this score to further improve its accuracy and geographical relevance. However, as we may expect the symptoms and patient behaviour to evolve with each Ebola outbreak, it is becoming increasingly important to create machine-learning predictive tools, which are able to better adapt to the changing statistics of future outbreaks.

## Supporting information

S1 Checklist STROBE Checklist. (DOCX)

S1 Fig Map of the distribution of EVD(+) admissions by section. (TIFF)

S2 Fig Triage protocol for suspect and probable EVD admissions used at the GOAL-Mathaska ETC. This triage protocol follows the WHO guidelines [7]
(TIFF)

S3 Fig Sensitivity and specificity of EVD triage score for various referral times. **(A)** Sensitivity and **(B)** specificity of the EVD triage score among patients arriving within 4 days of symptom onset (red), between 4 and 9 days of symptom onset (green) or after 9 days (blue).
(TIF)

S4 Fig Sensitivity and specificity of EVD triage score across malaria seasons. Sensitivity and specificity of the EVD triage score among patients over the entire study period (black, January-December), within the high malaria transmission season (red, May-October), and within the low malaria transmission season (orange, November-April). The area under the ROC curve for each population is indicated as AUC.
(TIFF)

S1 Table Complete data for the multivariate score to predict Ebola infection. (DOCX)

## References

[1] Weyer J, Grobbelaar A and Blumberg L, Ebola virus disease: history, epidemiology and outbreaks. Curr Infect Dis Rep, 2015 17(5): p. 480 10.1007/s11908-015-0480-y 25896751

[2] Centers for Disease Control and Prevention, Ebola Virus Disease Distribution Map, 17 Feb 2016.

[3] Bolkan HA, Bash-Taqi DA, Samai M, Gerdin M and von Schreeb J, Ebola and indirect effects on health service function in Sierra Leone. PLoS Curr, 2014 6. 10.1371/currents.outbreaks.0307d588df619f9c9447f8ead5b72b2d PMC4318968 25685617

[4] Awah PK, Boock AU and Kum KA, Ebola Virus Diseases in Africa: a commentary on its history, local and global context. Pan Afr Med J, 2015 22 Suppl 1: p. 18. 10.11694/pamj.supp.2015.22.1.6652 PMC4695518 26740846

[5] WHO Ebola Response Team, After Ebola in West Africa—Unpredictable Risks, Preventable Epidemics. N Engl J Med, 2016 375(6): p. 587–96. 10.1056/NEJMsr1513109 27509108

[6] Muyembe-Tamfum JJ, Kipasa M, Kiyungu C and Colebunders R, Ebola outbreak in Kikwit, Democratic Republic of the Congo: discovery and control measures. J Infect Dis, 1999 179 Suppl 1: p. S259–62. 9988192 10.1086/514302

[7] WHO. Case definition recommendations for Ebola or Marburg Virus Diseases. 2014 9 August].

[8] O'Shea MK, Clay KA, Craig DG, Matthews SW, Kao RL, Fletcher TE, et al, Diagnosis of Febrile Illnesses Other Than Ebola Virus Disease at an Ebola Treatment Unit in Sierra Leone. Clin Infect Dis, 2015 61(5): p. 795–8. 10.1093/cid/civ399 25991466 PMC7108066

[9] Lado M, Walker NF, Baker P, Haroon S, Brown CS, Youkee D, et al, Clinical features of patients isolated for suspected Ebola virus disease at Connaught Hospital, Freetown, Sierra Leone: a retrospective cohort study. Lancet Infect Dis, 2015 15(9): p. 1024–33. 10.1016/S1473-3099(15)00137-1 26213248

[10] Bah EI, Lamah MC, Fletcher T, Jacob ST, Brett-Major DM, Sall AA, et al, Clinical presentation of patients with Ebola virus disease in Conakry, Guinea. N Engl J Med, 2015 372(1): p. 40–7. 10.1056/NEJMoa1411249 25372658

[11] Levine AC, Shetty PP, Burbach R, Cheemalapati S, Glavis-Bloom J, Wiskel T, et al, Derivation and Internal Validation of the Ebola Prediction Score for Risk Stratification of Patients With Suspected Ebola Virus Disease. Ann Emerg Med, 2015 66(3): p. 285–293 e1. 10.1016/j.annemergmed.2015.03.011 25845607

[12] WHO Ebola Response Team, Ebola virus disease in West Africa—the first 9 months of the epidemic and forward projections. N Engl J Med, 2014 371(16): p. 1481–95. 10.1056/NEJMoa1411100 25244186 PMC4235004

[13] Schieffelin JS, Shaffer JG, Goba A, Gbakie M, Gire SK, Colubri A, et al, Clinical illness and outcomes in patients with Ebola in Sierra Leone. N Engl J Med, 2014 371(22): p. 2092–100. 10.1056/NEJMoa1411680 25353969 PMC4318555

[14] Qin E, Bi J, Zhao M, Wang Y, Guo T, Yan T, et al, Clinical Features of Patients With Ebola Virus Disease in Sierra Leone. Clin Infect Dis, 2015 61(4): p. 491–5. 10.1093/cid/civ319 25995207

[15] Hunt L, Gupta-Wright A, Simms V, Tamba F, Knott V, Tamba K, et al, Clinical presentation, biochemical, and haematological parameters and their association with outcome in patients with Ebola virus disease: an observational cohort study. Lancet Infect Dis, 2015 15(11): p. 1292–9. 10.1016/S1473-3099(15)00144-9 26271406

[16] Fitzpatrick G, Vogt F, Moi Gbabai OB, Decroo T, Keane M, De Clerck H, et al, The Contribution of Ebola Viral Load at Admission and Other Patient Characteristics to Mortality in a Medecins Sans Frontieres Ebola Case Management Centre, Kailahun, Sierra Leone, June-October 2014. J Infect Dis, 2015 212(11): p. 1752–8. 10.1093/infdis/jiv304 26002981 PMC4633764

[17] Zhang X, Rong Y, Sun L, Liu L, Su H, Zhang J, et al, Prognostic Analysis of Patients with Ebola Virus Disease. PLoS Negl Trop Dis, 2015 9(9): p. e0004113 10.1371/journal.pntd.0004113 26398207 PMC4580459

[18] de La Vega MA, Caleo G, Audet J, Qiu X, Kozak RA, Brooks JI, et al, Ebola viral load at diagnosis associates with patient outcome and outbreak evolution. J Clin Invest, 2015 125(12): p. 4421–8. 10.1172/JCI83162 26551677 PMC4665775

[19] Pagnoni F and Bosman A, Malaria kills more than Ebola virus disease. Lancet Infect Dis, 2015 15(9): p. 988–9. 10.1016/S1473-3099(15)00075-4 26116185 PMC7128846

[20] Plucinski MM, Guilavogui T, Sidikiba S, Diakite N, Diakite S, Dioubate M, et al, Effect of the Ebola-virus-disease epidemic on malaria case management in Guinea, 2014: a cross-sectional survey of health facilities. Lancet Infect Dis, 2015 15(9): p. 1017–23. 10.1016/S1473-3099(15)00061-4 26116183 PMC4669675

[21] Walker PG, White MT, Griffin JT, Reynolds A, Ferguson NM and Ghani AC, Malaria morbidity and mortality in Ebola-affected countries caused by decreased health-care capacity, and the potential effect of mitigation strategies: a modelling analysis. Lancet Infect Dis, 2015 15(7): p. 825–32. 10.1016/S1473-3099(15)70124-6 25921597 PMC4824180

[22] WHO, Clinical management of patients with viral haemorrhagic fever: a pocket guide for the front-line health workers. World Health Organization, Geneva, 2014.

[23] Roddy P, Colebunders R, Jeffs B, Palma PP, Van Herp M and Borchert M, Filovirus hemorrhagic fever outbreak case management: a review of current and future treatment options. J Infect Dis, 2011 204 Suppl 3: p. S791–5. 21987752 10.1093/infdis/jir297

[24] Kalton G and Kasprzyk D. Imputing missing survey responses in Proceedings of the section on survey research methods, American Statistical Association 1982.

[25] Royston P and Altman D, Regression using fractional polynomials of continuous covariates: parsimonious parametric modeling. Applied Statistics, 1994 43(3): p. 429–467.

[26] Harrell FE Jr., Lee KL and Mark DB, Multivariable prognostic models: issues in developing models, evaluating assumptions and adequacy, and measuring and reducing errors. Stat Med, 1996 15(4): p. 361–87. 10.1002/(SICI)1097-0258(19960229)15:4<361::AID-SIM168>3.0.CO;2-4 8668867

[27] Vogt F, Fitzpatrick G, Patten G, van den Bergh R, Stinson K, Pandolfi L, et al, Assessment of the MSF triage system, separating patients into different wards pending Ebola virus laboratory confirmation, Kailahun, Sierra Leone, July to September 2014. Euro Surveill, 2015 20(50). 10.2807/1560-7917.ES.2015.20.50.30097 26727011

[28] Dhillon RS, Srikrishna D, Garry RF and Chowell G, Ebola control: rapid diagnostic testing. Lancet Infect Dis, 2015 15(2): p. 147–8. 10.1016/S1473-3099(14)71035-7 25467648

[29] Rabelo I, Lee V, Fallah MP, Massaquoi M, Evlampidou I, Crestani R, et al, Psychological Distress among Ebola Survivors Discharged from an Ebola Treatment Unit in Monrovia, Liberia—A Qualitative Study. Front Public Health, 2016 4: p. 142 10.3389/fpubh.2016.00142 27458576 PMC4931229

[30] Zachariah R and Harries AD, The WHO clinical case definition for suspected cases of Ebola virus disease arriving at Ebola holding units: reason to worry? Lancet Infect Dis, 2015 15(9): p. 989–90. 10.1016/S1473-3099(15)00160-7 26213247

[31] Pittalis S, Fusco FM, Lanini S, Nisii C, Puro V, Lauria FN, et al, Case definition for Ebola and Marburg haemorrhagic fevers: a complex challenge for epidemiologists and clinicians. New Microbiol, 2009 32(4): p. 359–67. 20128442

[32] Petti S, Messano GA, Vingolo EM, Marsella LT and Scully C, The face of Ebola: changing frequency of haemorrhage in the West African compared with Eastern-Central African outbreaks. BMC Infect Dis, 2015 15: p. 564 10.1186/s12879-015-1302-4 26653293 PMC4676861

[33] The United States Central Intelligence Agency. World Fact book, Sierra Leone. 2015.

[34] Yan T, Mu J, Qin E, Wang Y, Liu L, Wu D, et al, Clinical characteristics of 154 patients suspected of having Ebola virus disease in the Ebola holding center of Jui Government Hospital in Sierra Leone during the 2014 Ebola outbreak. Eur J Clin Microbiol Infect Dis, 2015 34(10): p. 2089–95. 10.1007/s10096-015-2457-z 26223324

[35] Fallah MP, Skrip LA, Gertler S, Yamin D and Galvani AP, Quantifying Poverty as a Driver of Ebola Transmission. PLoS Negl Trop Dis, 2015 9(12): p. e0004260 10.1371/journal.pntd.0004260 26720278 PMC4697799

[36] Glynn JR, Age-specific incidence of Ebola virus disease. Lancet, 2015 386(9992): p. 432 10.1016/S0140-6736(15)61446-5 26251391

