# Supplementary material for: Predicting Ebola infection: A malaria-sensitive triage score for Ebola virus disease
Source: PLoS Negl Trop Dis. 2017 Feb 23;11(2):e0005356. doi: 10.1371/journal.pntd.0005356 (PMC5322888; doi:10.1371/journal.pntd.0005356)
Supplement: S1 Checklist — (DOCX) [file pntd.0005356.s001.docx]

STROBE Statement—checklist of items that should be included in reports of observational studies

|  | Item No. | Recommendation | Page  No. | Relevant text from manuscript |
| --- | --- | --- | --- | --- |
| **Title and abstract** | 1 | (*a*) Indicate the study’s design with a commonly used term in the title or the abstract | 1 | Title |
|  |  | (*b*) Provide in the abstract an informative and balanced summary of what was done and what was found | 3 | Abstract |
| Introduction | | | |  |
| Background/rationale | 2 | Explain the scientific background and rationale for the investigation being reported | 5 | Introduction |
| Objectives | 3 | State specific objectives, including any prespecified hypotheses | 6 | Introduction |
| Methods | | | |  |
| Study design | 4 | Present key elements of study design early in the paper | 7 | Methods (Study design) |
| Setting | 5 | Describe the setting, locations, and relevant dates, including periods of recruitment, exposure, follow-up, and data collection | 7 | Methods (Study design, patient referral, data collection) |
| Participants | 6 | (*a*) *Cohort study*—Give the eligibility criteria, and the sources and methods of selection of participants. Describe methods of follow-up | 8 | Methods (Cohort and inclusion criteria) |
|  |  | (*b*) *Cohort study*—For matched studies, give matching criteria and number of exposed and unexposed | n/a |  |
| Variables | 7 | Clearly define all outcomes, exposures, predictors, potential confounders, and effect modifiers. Give diagnostic criteria, if applicable | 7-9 | Methods (Data entry, Signs and Symptoms, Cohort and inclusion criteria, Data entry) |
| Data sources/ measurement | 8* | For each variable of interest, give sources of data and details of methods of assessment (measurement). Describe comparability of assessment methods if there is more than one group | 7-10 | Methods (Study design, patient referral, Data entry, Signs and Symptoms, Cohort and inclusion criteria, Primary data analysis, Calculation of the triage score and model validation) |
| Bias | 9 | Describe any efforts to address potential sources of bias | 8-10 | Methods (Data entry, Signs and Symptoms, Cohort and inclusion criteria, Primary data analysis, Calculation of the triage score and model validation) |
| Study size | 10 | Explain how the study size was arrived at | 7 | All EVD suspect patients limited to a single Ebola Treatment Center over the entire period of its operation. |

Continued on next page

| Quantitative variables | 11 | Explain how quantitative variables were handled in the analyses. If applicable, describe which groupings were chosen and why | 8-10 | Categorised variables are represented on a continuous scale in the supplement. The effect of categorisation was tested to be insignificant on the efficacy of the final scoring system |
| --- | --- | --- | --- | --- |
| Statistical methods | 12 | (*a*) Describe all statistical methods, including those used to control for confounding | 8-10 | Methods (Data entry, Signs and Symptoms, Cohort and inclusion criteria, Primary data analysis, Calculation of the triage score and model validation) |
|  |  | (*b*) Describe any methods used to examine subgroups and interactions | 8-10 | Methods (Primary data analysis, Calculation of the triage score and model validation) |
|  |  | (*c*) Explain how missing data were addressed | 8 | Methods (Cohort and inclusion criteria) |
|  |  | (*d*) *Cohort study*—If applicable, explain how loss to follow-up was addressed | 8 | Methods (Cohort and inclusion criteria)  (Dead on arrival or late transfers were excluded) |
|  |  | (*e*) Describe any sensitivity analyses | 8-10 | Methods (Primary data analysis, Calculation of the triage score and model validation) |
| Results | | | | |
| Participants | 13* | (a) Report numbers of individuals at each stage of study—eg numbers potentially eligible, examined for eligibility, confirmed eligible, included in the study, completing follow-up, and analysed | Various | Results (1) Figure 1 summarises cohort |
|  |  | (b) Give reasons for non-participation at each stage | *n/a* |  |
|  |  | (c) Consider use of a flow diagram | *n/a* |  |
| Descriptive data | 14* | (a) Give characteristics of study participants (eg demographic, clinical, social) and information on exposures and potential confounders | Various | Results (1) |
|  |  | (b) Indicate number of participants with missing data for each variable of interest | Various | Results (all) |
|  |  | (c) *Cohort study*—Summarise follow-up time (eg, average and total amount) | Various | Figure 1 summarises study period |
| Outcome data | 15* | *Cohort study*—Report numbers of outcome events or summary measures over time | Various | Figure 1 summarises study period vs the 2 outcomes of EVD(+) vs EVD(-) as well as malaria infection |
|  |  | *Case-control study—*Report numbers in each exposure category, or summary measures of exposure | *n/a* |  |
|  |  | *Cross-sectional study—*Report numbers of outcome events or summary measures | *n/a* |  |
| Main results | 16 | (*a*) Give unadjusted estimates and, if applicable, confounder-adjusted estimates and their precision (eg, 95% confidence interval). Make clear which confounders were adjusted for and why they were included | Various | Results (all) |
|  |  | (*b*) Report category boundaries when continuous variables were categorized | Various | Figure S3 show detailed information on category selection |
|  |  | (*c*) If relevant, consider translating estimates of relative risk into absolute risk for a meaningful time period | *n/a* |  |

| Other analyses | 17 | Report other analyses done—eg analyses of subgroups and interactions, and sensitivity analyses | Various | Results (all) |
| --- | --- | --- | --- | --- |
| Discussion | | | | |
| Key results | 18 | Summarise key results with reference to study objectives | 20-21 | Discussion |
| Limitations | 19 | Discuss limitations of the study, taking into account sources of potential bias or imprecision. Discuss both direction and magnitude of any potential bias | 22-23 | Discussion (limitations) |
| Interpretation | 20 | Give a cautious overall interpretation of results considering objectives, limitations, multiplicity of analyses, results from similar studies, and other relevant evidence | 23 | Discussion (limitations and conclusion) |
| Generalisability | 21 | Discuss the generalisability (external validity) of the study results | 22-23 | Discussion (limitations and conclusion) |
| Other information | |  | | |
| Funding | 22 | Give the source of funding and the role of the funders for the present study and, if applicable, for the original study on which the present article is based | 10 | Also provided in manuscript submission database |

*Give information separately for cases and controls in case-control studies and, if applicable, for exposed and unexposed groups in cohort and cross-sectional studies.

**Note:** An Explanation and Elaboration article discusses each checklist item and gives methodological background and published examples of transparent reporting. The STROBE checklist is best used in conjunction with this article (freely available on the Web sites of PLoS Medicine at http://www.plosmedicine.org/, Annals of Internal Medicine at http://www.annals.org/, and Epidemiology at http://www.epidem.com/). Information on the STROBE Initiative is available at www.strobe-statement.org.
