# Supplementary figures and images for: Predicting Ebola infection: A malaria-sensitive triage score for Ebola virus disease
Source: PLoS Negl Trop Dis. 2017 Feb 23;11(2):e0005356. doi: 10.1371/journal.pntd.0005356 (PMC5322888; doi:10.1371/journal.pntd.0005356)

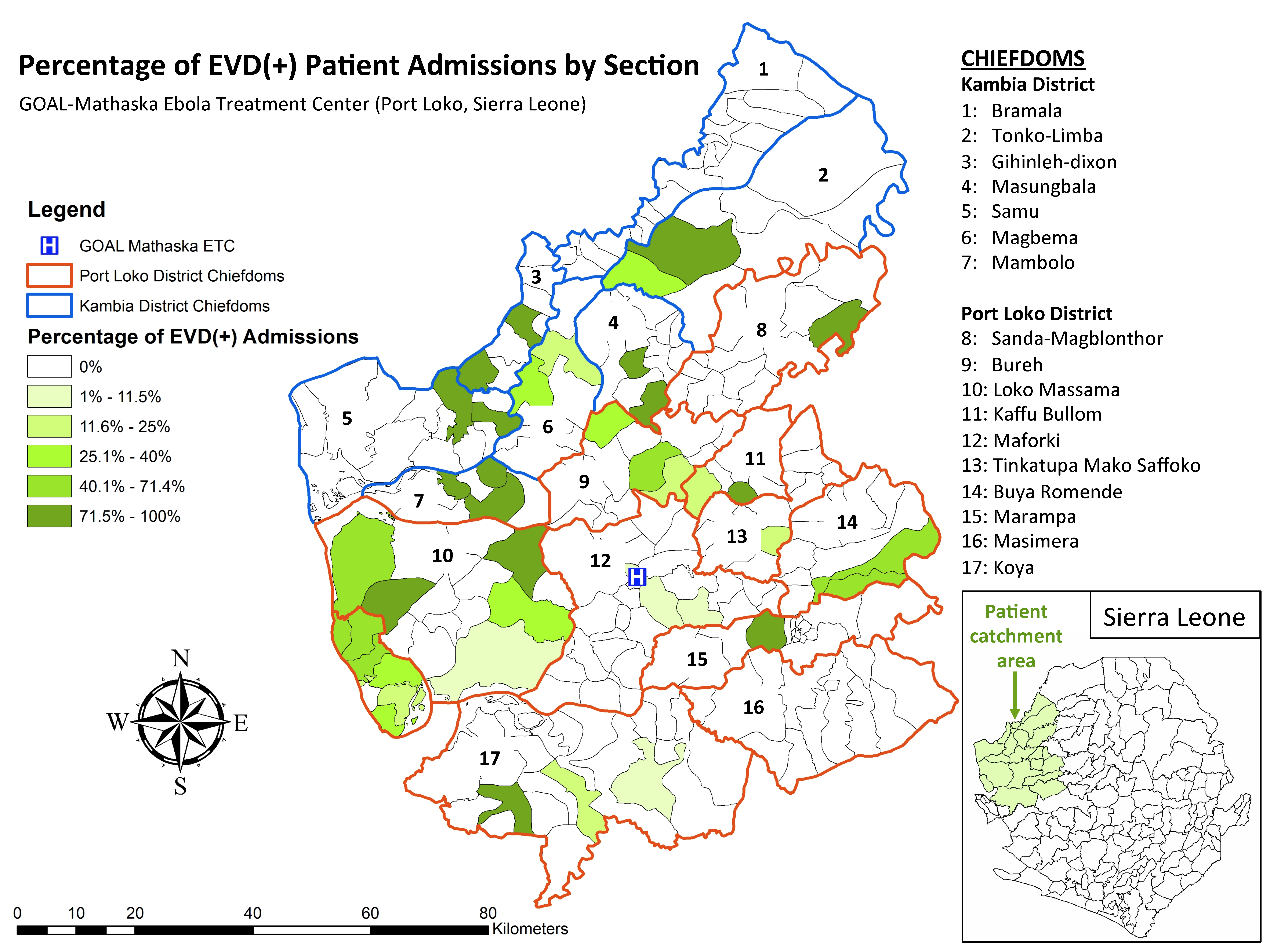

Supplement: S1 Fig — (TIFF) [file pntd.0005356.s002.tiff]

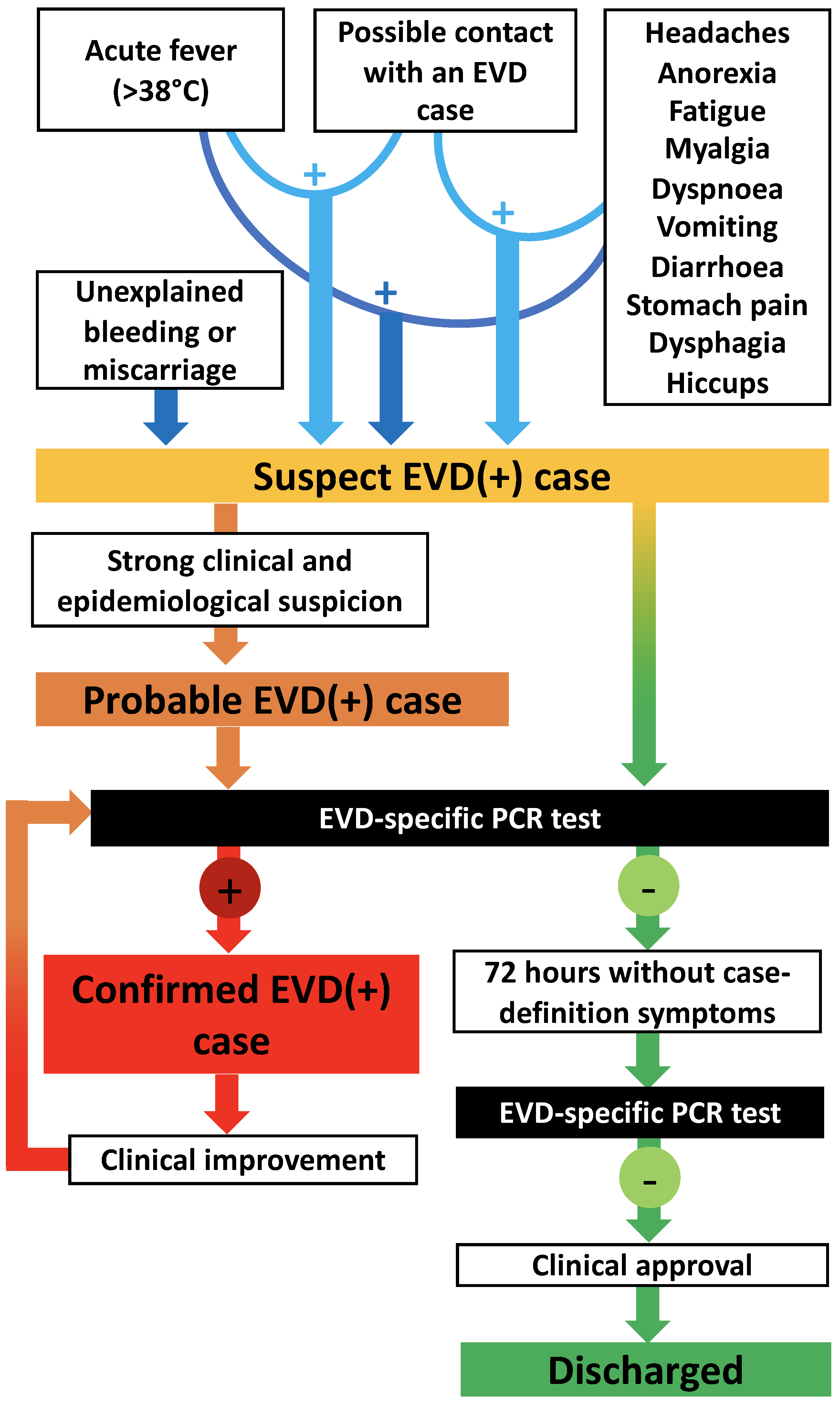

Supplement: S2 Fig — This triage protocol follows the WHO guidelines [7] (TIFF) [file pntd.0005356.s003.tiff]

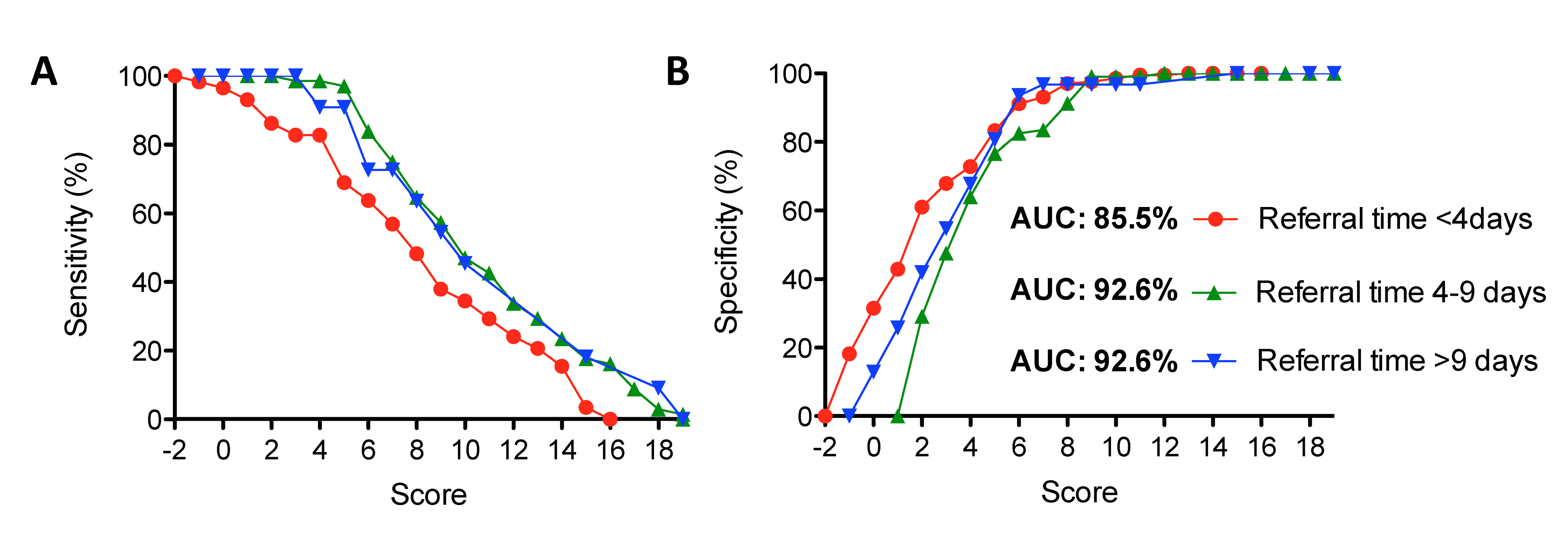

Supplement: S3 Fig — (A) Sensitivity and (B) specificity of the EVD triage score among patients arriving within 4 days of symptom onset (red), between 4 and 9 days of symptom onset (green) or after 9 days (blue). (TIF) [file pntd.0005356.s004.tif]

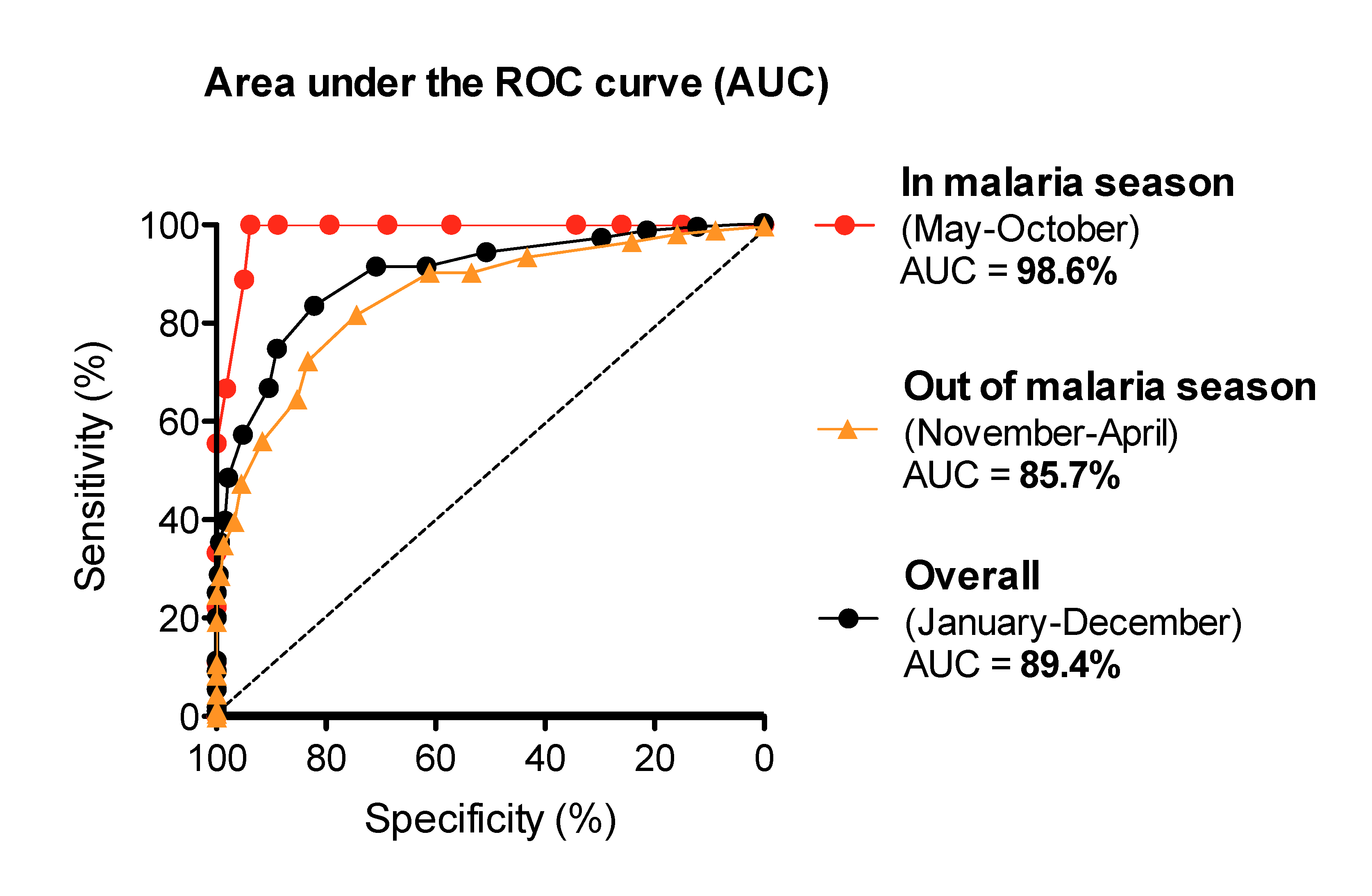

Supplement: S4 Fig — Sensitivity and specificity of the EVD triage score among patients over the entire study period (black, January-December), within the high malaria transmission season (red, May-October), and within the low malaria transmission season (orange, November-April). The area under the ROC curve for each population is indicated as AUC. (TIFF) [file pntd.0005356.s005.tiff]
