# Supplementary material for: Predicting Ebola infection: A malaria-sensitive triage score for Ebola virus disease
Source: PLoS Negl Trop Dis. 2017 Feb 23;11(2):e0005356. doi: 10.1371/journal.pntd.0005356 (PMC5322888; doi:10.1371/journal.pntd.0005356)
Supplement: S1 Table — (DOCX) [file pntd.0005356.s006.docx]

**Table S1**

| **Multivariate predictors for the risk of EVD infection** | **OR** | **Coefficient** | **Std Error** | **p value** | **CI95%** | |
| --- | --- | --- | --- | --- | --- | --- |
| **Possible contact with Ebola** | 19.4 | 3.0 | 6.5 | 0.000 | 10.1 | 37.4 |
| **Days since 1^st^ symptom = 4-9 days** | 3.7 | 1.3 | 1.1 | 0.000 | 2.1 | 6.7 |
| **Conjunctivitis** | 7.2 | 2.0 | 2.3 | 0.000 | 3.8 | 13.6 |
| **Diarrhoea** | 3.8 | 1.3 | 1.1 | 0.000 | 2.1 | 6.8 |
| **Dysphagia** | 2.2 | 0.8 | 0.9 | 0.034 | 1.1 | 4.8 |
| **Haemorrhage** | 2.9 | 1.0 | 1.5 | 0.036 | 1.1 | 8.0 |
| **Temperature >38°C** | 1.8 | 0.6 | 0.5 | 0.044 | 1.0 | 3.1 |
| **Myalgia** | 0.5 | -0.8 | 0.1 | 0.012 | 0.2 | 0.8 |
| ***Intercept constant*** | 0.4 | -3.3 | 0.01 | 0.000 | 0.0 | 0.1 |
